# Impact of Cutting Parameters and Tool Type on Surface Finish in MQL Turning of Inconel 625

**DOI:** 10.3390/ma18194617

**Published:** 2025-10-06

**Authors:** Magdalena Machno, Wojciech Zębala, Emilia Franczyk

**Affiliations:** 1 Department of Rail Vehicles and Transport, Faculty of Mechanical Engineering, Cracow University of Technology, 31-155 Cracow, Poland; 2 Department of Production Engineering, Faculty of Mechanical, Cracow University of Technology, 31-155 Cracow, Poland; wojciech.zebala@pk.edu.pl (W.Z.); emilia.franczyk@pk.edu.pl (E.F.)

**Keywords:** Inconel 62 superalloy, turning, minimum quantity lubrication (MQL), surface roughness, cutting parameters, chip type

## Abstract

Inconel 625 is a nickel-based superalloy widely applied in aerospace and energy sectors due to its high strength and corrosion resistance. However, its poor machinability remains a significant challenge in precision manufacturing. This study investigates the influence of tool geometry and cutting parameters on surface roughness of Inconel 625 during turning operations under the minimum quantity lubrication (MQL) conditions. Experiments were carried out using three types of cutting inserts with distinct chip breaker geometries while systematically varying the cutting speed, feed rate, and depth of cut. The results were statistically analyzed using analysis of variance (ANOVA) to determine the significance of individual factors. The findings reveal that both the type of cutting insert and the process parameters have a considerable effect on surface roughness, which is the key output examined in this study. Cutting forces and chip type were examined to provide complementary insights and improve understanding of the observed relationships. Based on the results, an optimal set of cutting data was proposed to achieve a required surface roughness during the turning of Inconel 625 with MQL. Furthermore, a practical algorithm was developed to support the selection of cutting parameters in industrial applications. Analysis of the results showed that a cutting insert with a 0.4 mm corner radius achieved the required surface finish (Rz ≤ 0.4 µm). Furthermore, the analysis revealed a significant effect of the thermal properties of Inconel 625 on machining results and chip geometry.

## 1. Introduction

Nickel–chromium superalloys, such as Inconel 625, are widely used in aerospace, nuclear, marine, and chemical industries due to their high-temperature strength, corrosion resistance, and toughness [1,2,3]. Alloy 625 is particularly valued for its excellent weldability and corrosion resistance, making it a versatile material for demanding applications [4,5]. Inconel 625 is a single-phase austenitic nickel–chromium superalloy whose exceptional properties arise from solid-solution strengthening by Cr, Mo, Nb, and Fe, complemented by precipitation hardening from intermetallic phases such as NbC, δ, and Laves [2,3,6]. The alloy is supplied as sheets, bars, tubes, or coatings on cheaper substrates to enhance performance [7,8], and can be processed using conventional methods such as casting, forming, machining, and welding, as well as emerging additive manufacturing techniques [1,9,10]. Inconel 625 is also suitable for electrical discharge machining, but due to its low thermal conductivity, chemical reactivity with tools, high hardness, and low elastic modulus, conventional machining remains challenging and subject to continuous optimization [11,12,13,14,15]. To achieve the required surface quality, finishing processes such as grinding, abrasive-flow machining, chemical and electrochemical polishing, or combined methods are often applied [16,17].

One of the most important technological parameters that influences the selection of machining process parameters is the quality of the machined surface. Surface quality analysis is most often performed by selecting process parameters to obtain the appropriate surface roughness value (Ra or Rz). The machining of Inconel 625 is considerably influenced by its mechanical and thermophysical properties, which makes the process a substantial technological challenge, particularly with regard to the surface quality of the workpiece. Surface roughness during the cutting process is determined by phenomena such as the following [18,19]:

- High hot strength: high forces at elevated temperatures—more heat and unstable cutting;
- High thermal expansion: dimensional instability—vibration and chatter marks;
- High work hardening: increased cutting forces—smearing and tearing of the surface;
- Low thermal conductivity: localized heat accumulation—tool wear and plastic deformation.

The low thermal conductivity of Inconel 625 causes cutting temperatures at the tool rake face to reach up to 1200 °C. Its high strength, hardness, impact resistance, and tendency toward work hardening result in elevated cutting forces and difficulties in chip breaking. Moreover, the chemical affinity of the alloy and the hardness of carbides accelerate tool wear, thereby affecting both surface quality and workpiece geometry [5]. Due to its high thermal resistance and hot-hardening tendency, heat accumulates in the cutting zone, leading to tool overheating and loss of cutting efficiency. This results in poor surface finish and increased energy consumption. Consequently, careful selection of cutting parameters and the use of cooling–lubricating strategies, such as minimum quantity lubrication (MQL), are essential [20].

The machining of nickel–chromium superalloys remains a technological challenge, as processes must be both efficient and cost-effective while ensuring acceptable surface quality. As reported in [3], major issues in turning Inconel 625 and 718 include the high specific cutting energy (SCE) and rapid work hardening of the surface layer. Surface roughness is primarily influenced by cutting parameters such as cutting speed, feed rate, and depth of cut [21,22]. Higher feed rates increase roughness, whereas optimal conditions were reported for *vc* = 45 m/min, *f* = 0.05 mm/rev, and *ap* = 0.2 mm.

Tool wear studies [4] identified abrasion, adhesion, and oxidation as the dominant wear mechanisms. At higher cutting conditions, tool chipping, tipping, and even fracture were observed. Optimal parameters were reported as *vc* = 60 m/min, *f* = 0.1 mm/rev, and *ap* = 0.5 mm. The application of coolants, particularly MQL and nanofluid-MQL, significantly improves chip evacuation, heat dissipation, tool life, and surface roughness [22,23].

Comparative investigations of nickel-based alloys (Inconel 718, Inconel 625, Monel 400) confirmed considerable differences in tool life arising from their distinct thermal and mechanical properties [5]. Inconel 718, characterized by lower thermal conductivity and higher hardness, exhibited shorter tool life compared with Inconel 625 and Monel 400 under identical cutting conditions.

In summary, the unique thermophysical and mechanical properties of nickel–chromium superalloys make their machining highly demanding. Heat generation in the cutting zone remains the primary challenge, and further experimental research is required to optimize cutting parameters and to achieve predictable surface quality.

Nickel–chromium superalloys such as Inconel 625 remain among the most difficult materials to machine. In response, this study focuses on the turning of Inconel 625 using the minimum quantity lubrication (MQL), focusing on the effects of cutting speed, feed, and depth of cut on surface roughness (Rz). The results are compared with dry turning tests presented in [24], with particular attention to achieving Rz ≤ 0.4 µm. The variable turning parameters included cutting speed, feed per revolution, and the depth of cut. The study analyzed the effect of these variable parameters on the obtained surface roughness defined by the Rz parameter. The analysis results will provide information on the characteristics of the turned surface of Inconel 625 and the effect of the thermal properties of this superalloy on the turning process.

## 2. Materials and Methods

Experimental turning tests of Inconel 625 were performed at Cracow University of Technology on a 50 mm diameter shaft. This alloy was chosen due to its extensive use in aerospace, energy, and defense applications, as well as its known machining challenges. Table 1 presents its chemical composition, and Table 2 lists selected mechanical and thermophysical properties, which were key factors considered during the analysis of the turning results.

Turning tests were conducted using minimum quantity lubrication (MQL). Effective cooling is crucial when machining this superalloy to reduce tool overheating, minimize built-up edge formation, and improve turning performance. A total of three test campaigns were carried out, each using one of three different cutting inserts. The selected cutting inserts are designed for machining difficult-to-machine materials, such as nickel–chromium superalloys. These cutting inserts are frequently used in industrial materials processing, which also determined their selection for experimental testing. The following cutting inserts were used in the experimental tests:

- CNMG120408-MF4, grade: TS2500—corner radius RE = 0.8 mm, PVD coated, ISO-P carbide for turning, with chip breaker designation MF4 referred to as TS in this study;
- CNMG120404-MF1, grade: CP200—corner radius RE = 0.4 mm, PVD coated, ISO-P carbide for turning, with chip breaker designation MF1 referred to as CP;
- CNMG120404-MF1, grade: TH1000—corner radius RE = 0.4 mm, PVD coated, ISO-P carbide for turning, with chip breaker designation MF1 referred to as TH.

**Figure 1:**
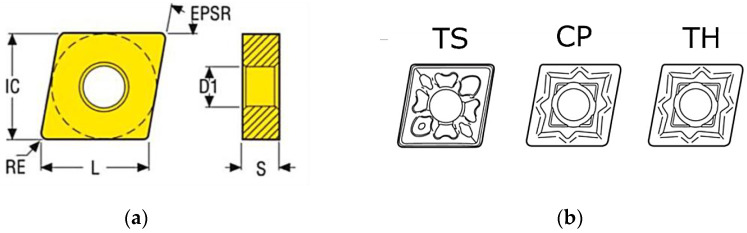
(**a**) Scheme of the cutting insert geometry; (**b**) type of chip breaker.

The cutting inserts used were manufactured by SECO. Common features of all inserts include an included angle (EPSR) of 80°, cutting edge length (L) of 12.90 mm, fixing hole diameter (D1) of 5.1 mm, inscribed circle diameter (IC) of 12.7 mm, and insert thickness (S) of 4.76 mm (Figure 1) [5]. Each test campaign was repeated three times.

Turning tests were performed on a Knuth Masterturn 400 × 1000 lathe (KNUTH, Wasbek, Germany) with a tool approach angle of 95° and a cutting edge angle of 6°. The cutting insert was mounted in a PCLNR 2020K-12 holder. Cutting forces were measured using a KISTLER 9257B piezoelectric dynamometer (Kistler Group, Winterthur, Switzerland) connected via a KISTLER 5070B charge amplifier (Kistler Group, Winterthur, Switzerland) to a PC system running the DynoWare software (Version 2825A, Kistler Group, Winterthur, Switzerland). The feed force *Ff* was analyzed in detail due to its significant impact on surface roughness.

Experiments followed a Taguchi experimental plan with three variable turning parameters: cutting speed (*vc* = 70, 100, and 130 m/min), feed per revolution (*f* = 0.077, 0.115, and 0.154 mm/rev), and depth of cut (*ap* = 0.1, 0.5 mm). Each test campaign was repeated three times. Table 3 summarizes the experimental plan and the average Rz surface roughness values obtained with the three cutting inserts.

**Figure 2:**
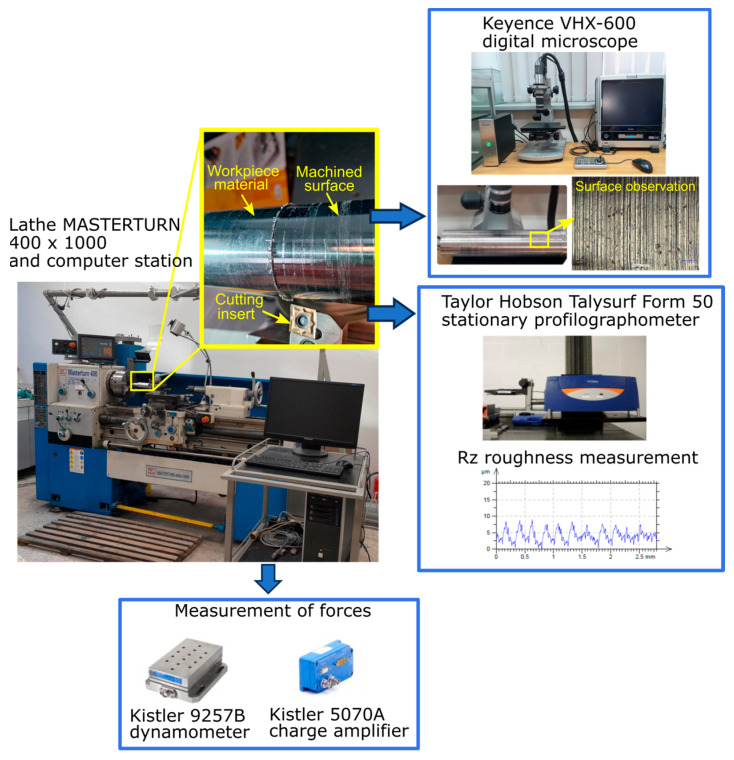
The scheme of experimental setup and measurement procedure.

The influence of variable turning parameters on surface roughness Rz was investigated. Rz was selected over Ra to provide a more detailed assessment of surface quality. For surfaces after finishing machining of difficult-to-cut alloys such as Inconel 625, it is recommended to use the Rz parameter, as it accurately reflects the presence of individual deeper scratches and grooves typical of this type of machining, which can significantly affect fatigue life and surface layer properties. The Ra parameter could underestimate the quality assessment, masking local irregularities that are critical for the performance of components made from such alloys. Measurements were performed using a Taylor Hobson Talysurf Intra 50 profilometer (Taylor Hobson, Leicester, UK) with a 2 µm rounding radius in a measurement tip and a Gaussian filter (*λ_c_* = 0.8 mm) according to [25] For each surface, three 4 mm measurements were taken, and the mean Rz value was used for analysis. Figure 2 shows the experimental setup and measurement procedure.

The main objective of this study is to analyze the surface quality obtained during turning under minimum quantity lubrication (MQL). Additionally, the study aims to identify the optimal cutting parameters and insert type to achieve a surface roughness of Rz ≤ 0.4 µm. The results were compared with dry turning, as reported in [24]. An analysis of variance (ANOVA) was conducted to evaluate the influence of variable turning parameters on surface roughness Rz, using the Minitab 22 statistical software (Minitab LLC., State College, PA, USA). Additionally, the geometry of the chip obtained for various sets of parameters was investigated. While certain studies focus on detailed chip formation mechanisms [26], the present work considers only general features to identify empirical trends. Microscopic observations of the chips and machined surfaces were performed using a Keyence VHX-600 digital microscope (Keyence, Osaka, Japan).

## 3. Results

### Analysis of the Influence of Parameters on Rz Surface Roughness and Selection of the Most Effective Conditions

**Figure 3:**
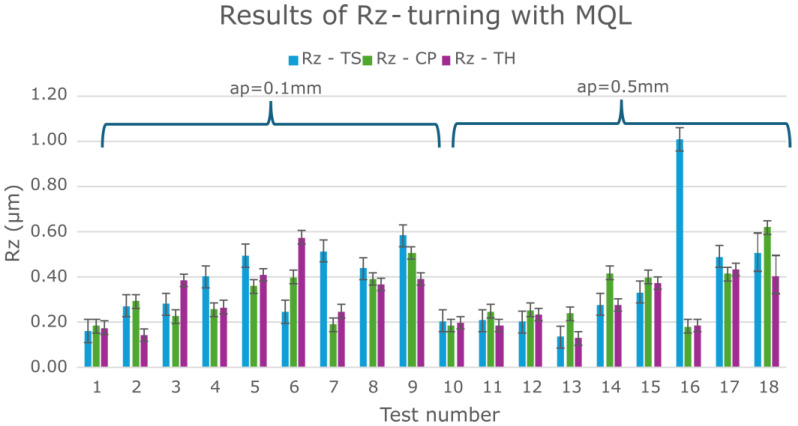
Results for all Inconel 625 turning tests using the MQL cooling.

Initially, all results obtained from the Taguchi experimental plan for the three cutting inserts (TS, CP, TH) under MQL were analyzed (Figure 3). Surface roughness values of Rz ≤ 0.4 µm were considered acceptable. For turning with a depth of cut of 0.1 mm (tests 1–9) and 0.5 mm (tests 10–18), most Rz values were below 0.6 µm.

Table 4 shows the results of the mean values of Rz and the standard deviation values.

**Figure 4:**
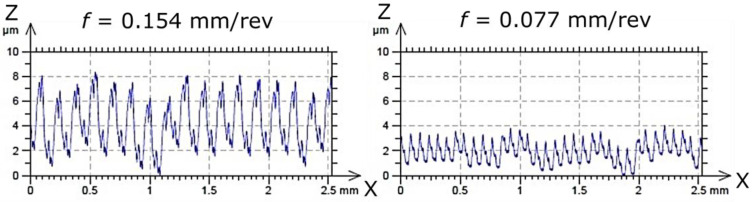
Comparison of surface profiles after turning with the use of extreme values of feed rate (*f*) (turning parameters: *vc* = 70 m/min, *ap* = 0.1 mm, and using the TH tool).

A clear effect of feed per revolution (*f*) was observed: smaller feed values resulted in better surface quality. Additionally, the TS cutting insert in test 16 exhibited a significantly higher Rz (≈1 µm), and, in several other experimental tests (4, 5, 7, 8, 9, 17), Rz values were higher compared to the CP and TH cutting inserts. These tests were conducted at the smallest feed per revolution (0.077 mm/rev). It should be emphasized that lower feed rates improve surface quality. Figure 4 shows the surface profiles after the turning process with extreme values of feed per revolution to illustrate the difference in the obtained surface quality.

**Figure 5:**
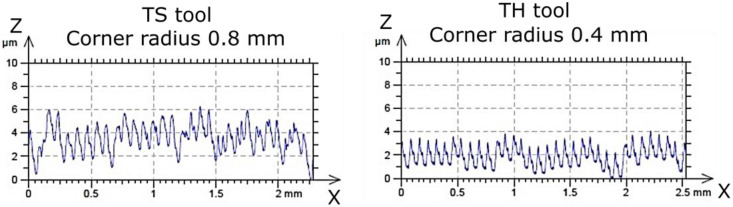
Comparison of surface profiles after turning with cutting inserts with different corner radius (turning parameters: *vc* = 70 m/min, *f* = 0.077 mm/rev, *ap* = 0.1 mm).

Moreover, the TS cutting insert has a larger corner radius (0.8 mm) compared to CP and TH (0.4 mm) and features a different chip breaker design. This analysis demonstrates that the type and geometry of the cutting insert significantly influence the resulting surface roughness. To illustrate the difference in the quality of the machined surface obtained after turning with a cutting insert with a corner radius of 0.4 mm and 0.8 mm, the corresponding surface profiles are presented in Figure 5.

Therefore, the test results show that cutting insert geometry (specifically, its nose radius) significantly improves surface roughness. A lower feed rate also improves surface roughness; in these tests, *f* = 0.077 mm/rev. Summarizing this part of the analysis, we can conclude that cutting insert geometry, nose radius, and feed per revolution strongly determine surface roughness. Similar conclusions were drawn based on other research results included in the Section 1.

**Figure 6:**
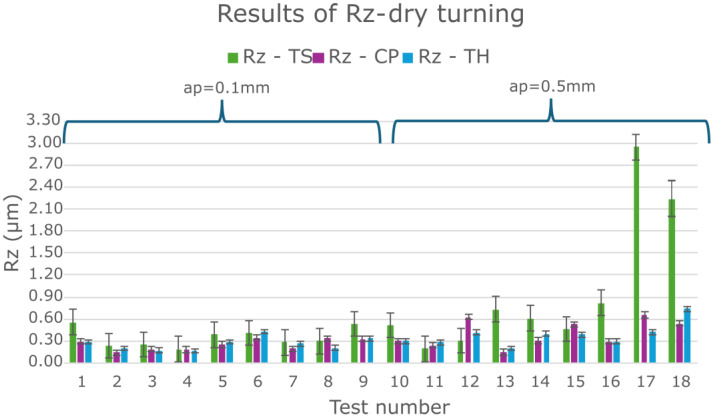
Results for all Inconel 625 turning tests under dry machining.

In order to better analyze the influence of insert type on Rz surface roughness, the MQL turning results were compared with the dry turning results under the same experimental plan (Figure 6). Analysis of these results also shows that the TS tool produced some Rz roughness results that deviated significantly from the corresponding Rz results for the CP and TH tools. Table 5 presents the average Rz values along with their corresponding standard deviations.

It can be concluded that certain features of the cutting insert significantly influence surface roughness. Therefore, turning of Inconel 625 is more effective when using CP and TH inserts, which have a 0.4 mm corner radius and a manufacturer-designated MF1 chip breaker. This conclusion is primarily based on the observation that the TS insert produced higher surface roughness values even at the lowest feed per revolution. Consequently, further analysis focused on the results obtained with the CP and TH cutting inserts.

For turning tests with the CP and TH cutting inserts, an analysis of variance (ANOVA) was performed using the Minitab statistical software 22. Resulting approximation functions are listed below:

- The CP cutting insert (Equation (1)):

Rz _CP(*vc*,*f*,*ap*) = 0.3203 − 0.0882 *vc_70* + 0.0248 *vc_100* + 0.0634 *vc_130* − 0.1138 *f_0.077* + 0.0336 *f_0.115* + 0.0802 *f_0.154* − 0.0086 *ap_0.1* + 0.0086 *ap_0.5*, (1)

- The TH cutting insert (Equation (2)):

Rz_TH(*vc*,*f*,*ap*) = 0.2992 − 0.0782 *vc_70* + 0.0390 *vc_100* + 0.0393 *vc_130* − 0.0987 *f_0.077* + 0.0038 *f_0.115* + 0.0949 *f_0.154* + 0.0300 *ap_0.1* − 0.0300 *ap_0.5*. (2)

A general linear model was selected for the regression analysis. The generated coefficients were considered significant at *p*-value ≤ 0.05. Regression results indicated that the depth of cut has a minimal effect on Rz surface roughness. Table 6 presents the ANOVA results (DF—total degrees of freedom, Adj SS—adjusted sums of squares, Adj MS—adjusted mean squares). Moreover, the fitted functional models have statistical coefficients R-sq (R-squared) and R-sq(adj) (adjusted R-squared) at a high level above 70% and above 60%, respectively (see Table 7).

**Figure 7:**
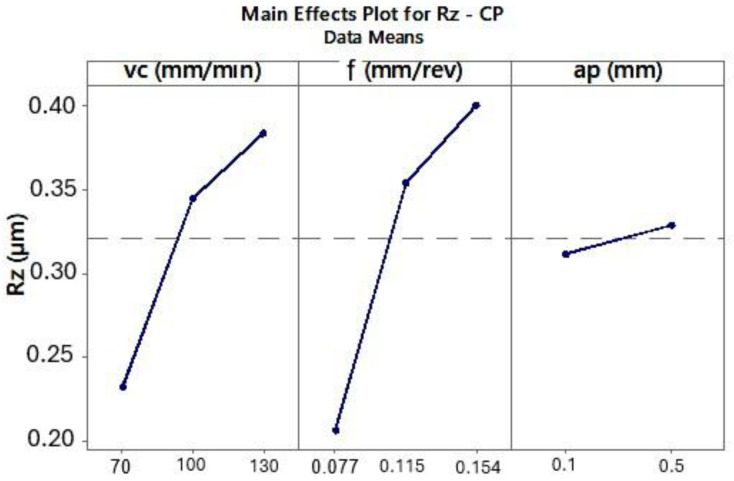
The influence of analyzed variables on the mean value of Rz for the CP tool.

**Figure 8:**
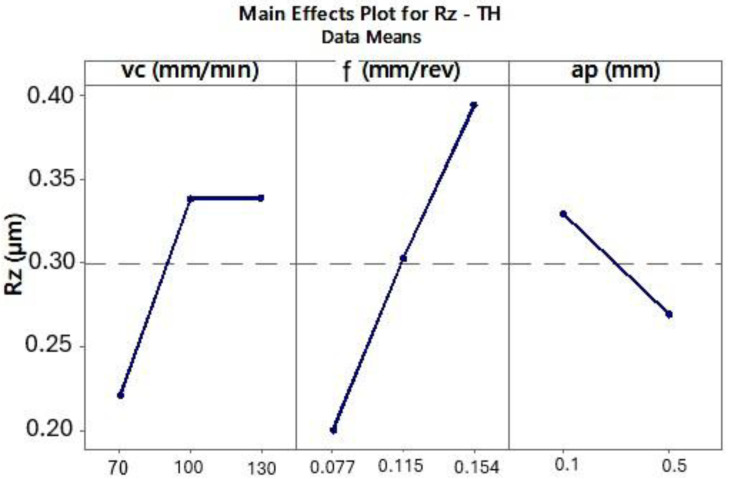
The influence of analyzed variables on the mean value of Rz for the TH tool.

The ANOVA results for the CP and TH cutting inserts showed that surface roughness Rz decreases with decreasing cutting speed (*vc*) and lower feed per revolution (*f*) (Figure 7 and Figure 8).

**Figure 9:**
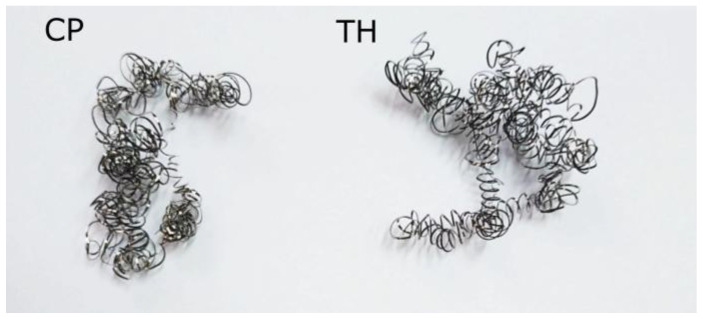
Chip photos—turning parameters: *vc* = 130 m/min, *f* = 0.115 mm/rev, *ap* = 0.1 mm, using CP and TH tools.

Inconel 625 exhibits specific thermal properties that complicate its machining. The material heats up more slowly due to its low thermal conductivity and high temperature resistance [27]. Consequently, higher cutting speeds can generate more heat, which is dissipated into both the cutting tool and the chips. The hotter chips tend to adhere more easily to the insert contact surface. In tests at a cutting speed of 130 m/min, the chips were more twisted, reflecting their intense heating during turning (Figure 9). At higher cutting speeds, some chips adhered to the tool and required mechanical removal from the cutting insert, confirming that a significant portion of the heat generated in the cutting zone is absorbed by the chips.

Other studies [22] recommend using lower cutting speeds (*vc*), preferably below 100 m/min, for machining this superalloy. Due to the specific thermophysical properties of Inconel 625, the experimental results also showed that lower cutting speeds led to reduced Rz surface roughness values. Moreover, decreasing the feed per revolution resulted in lower surface roughness, which is a typical relationship in turning processes.

Next, the influence of turning parameters on the feed force component *Ff* was analyzed for the CP and TH cutting inserts, also using ANOVA. For the analyzed *Ff* force and the selected inserts, the following approximating functions were obtained:

- The CP tool (Equation (3)):

Ff_CP(*vc*,*f*,*ap*) = 56.95 + 4.52 *vc_70* − 1.66 *vc_100* − 2.86 *vc_130* − 14.74 *f_0.077* + 0.12 *f_0.115* + 14.62 *f_0.154* + 24.43 *ap_0.1* − 24.43 *ap_0.5*, (3)

- The TH tool (Equation (4)):

Ff_TH(*vc*,*f*,*ap*) = 56.30 + 3.65 *vc_70* − 0.74 *vc_100* − 2.92 *vc_130* − 9.07 *f_0.077* + 1.12 *f_0.115* + 7.95 *f_0.154* + 25.97 *ap_0.1* − 25.97 *ap_0.5*. (4)

A linear model was adopted for the regression analysis. The generated coefficients were considered significant at *p* ≤ 0.05. Regression results indicated that cutting speed has a minimal effect on the feed force component *Ff* for both inserts. Table 8 presents the ANOVA results. Furthermore, the fitted regression models exhibited high statistical reliability, with R-sq and adjusted R-sq values exceeding 90% (Table 9).

**Figure 10:**
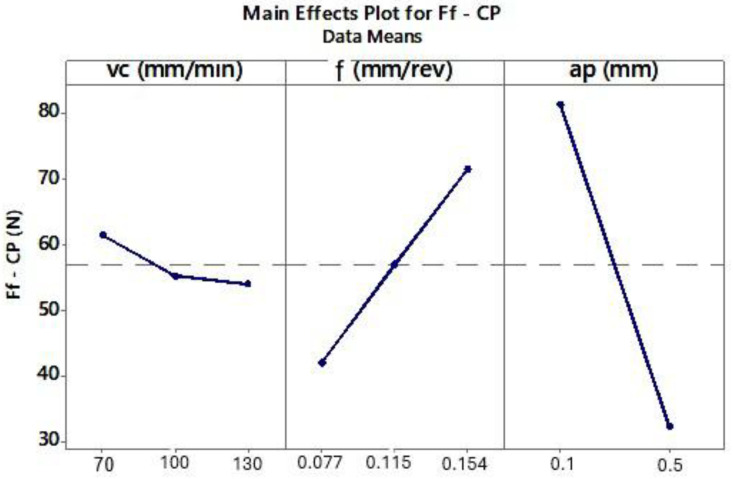
The influence of analyzed variables on the mean value of *Ff* for the CP tool.

**Figure 11:**
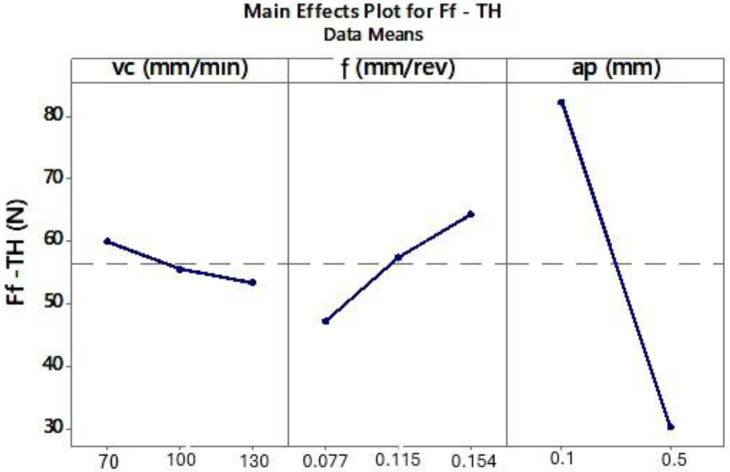
The influence of analyzed variables on the mean value of *Ff* for the TH tool.

Analyzing the effect of cutting parameters on the feed force component Ff during turning of Inconel 625 (Figure 10 and Figure 11), lower Ff values were observed at lower feed rates. Previous analysis also showed that smaller feeds resulted in improved surface roughness. ANOVA results indicated that lower Ff forces occurred at a depth of cut of 0.5 mm. However, the surface roughness values for depths of 0.1 mm and 0.5 mm were similar for the corresponding tests (Figure 3). Therefore, to determine the most effective depth of cut, the chips produced during turning were analyzed. The shape and type of chips influence machining efficiency, ease of chip separation from the tool–workpiece interface, and the quality of the machined surface.

The analysis of chip shape and type was carried out under the following conditions:

- The applied cutting speed;
- The depths of cut of 0.1 mm and 0.5 mm;
- The TS, CP, and TH inserts.

**Figure 12:**
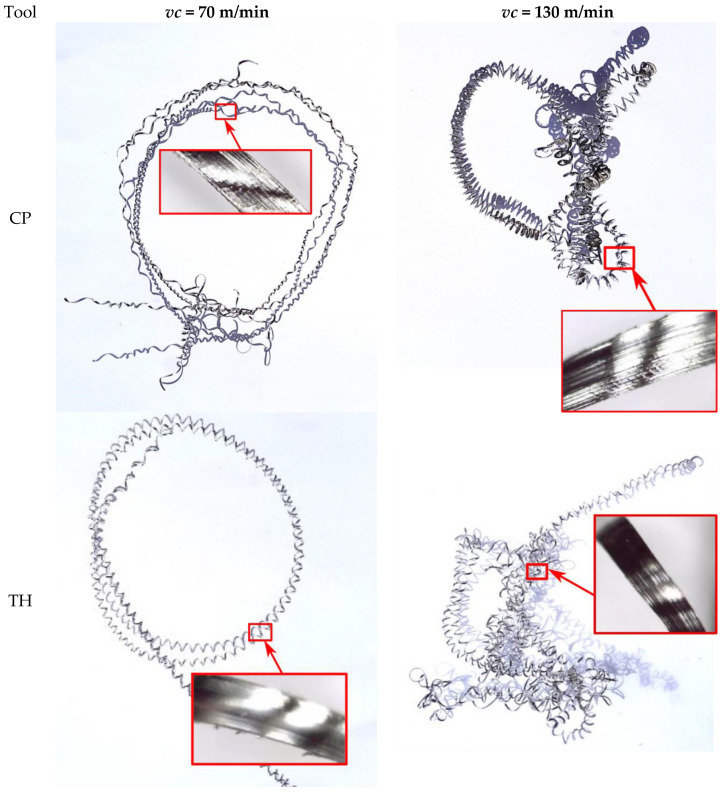
Chips for the extreme cutting speeds (*vc*) used, feed rate 0.077 mm/rev, and depth of cut 0.1 mm for the CP and TH cutting inserts.

As shown in Figure 12, the chips obtained at a depth of cut of 0.1 mm can be classified as long. At a cutting speed of *vc* = 70 m/min, the chips were less spiral and less twisted compared to those produced at *vc* = 130 m/min. Chips at *vc* = 70 m/min remained more extended, facilitating their separation from the tool edge. In contrast, at *vc* = 130 m/min, the chips were highly twisted and more spiral in shape. During turning, these chips twisted strongly at the tool–workpiece contact zone and rubbed against the machined surface, making them more difficult to detach, often requiring manual removal. Consequently, the Rz surface roughness parameter for the maximum cutting speed *vc* = 130 m/min could be higher due to this effect.

It can be assumed that during turning of Inconel 625, long, less spiral chips favor improved surface quality and are easier to detach during machining. In this case, there is less chance of damaging the machined surface due to the formed chip.

**Figure 13:**
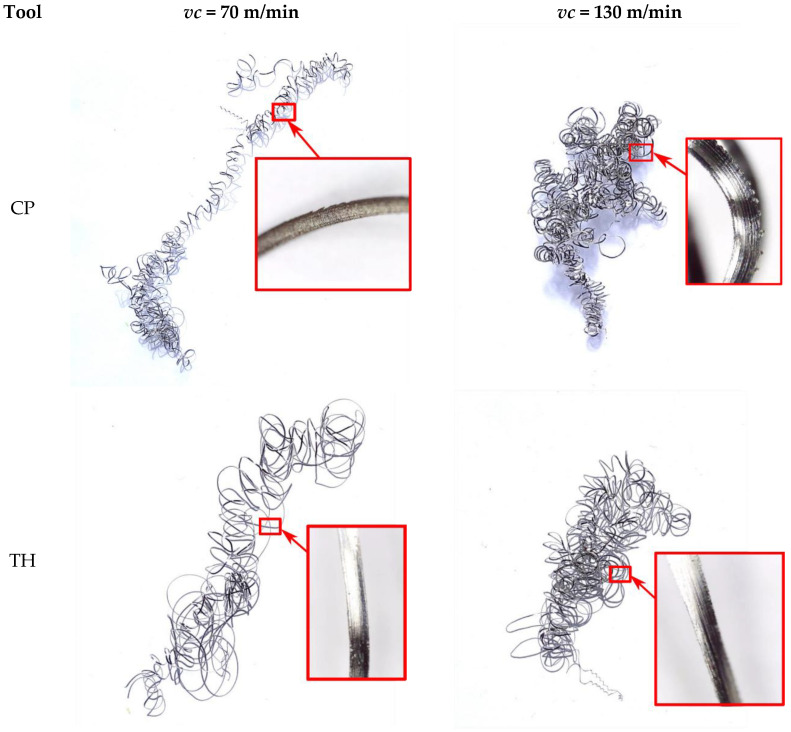
Chips for the extreme cutting speeds (*vc*) used, feed rate 0.077 mm/rev, and depth of cut 0.5 mm for the CP and TH cutting inserts.

Analyzing the chips produced at a depth of cut of 0.5 mm for the CP and TH cutting inserts, the chip shape was similar for both extreme cutting speeds (70 m/min and 130 m/min) (Figure 13). These chips were highly twisted, leading to accumulation in the tool zone and making them difficult to detach from the tool–workpiece interface. Additionally, at a cutting speed of 130 m/min, some chips adhered to the insert in several tests. The low thermal conductivity of Inconel 625 significantly influenced the cutting conditions in these experiments. The strong twisting of chips at *vc* = 130 m/min could result from their increased heating during turning. One potential solution in future tests could be more intensive cooling of the cutting zone to reduce heat transfer to both the chips and the cutting tool material.

**Figure 14:**
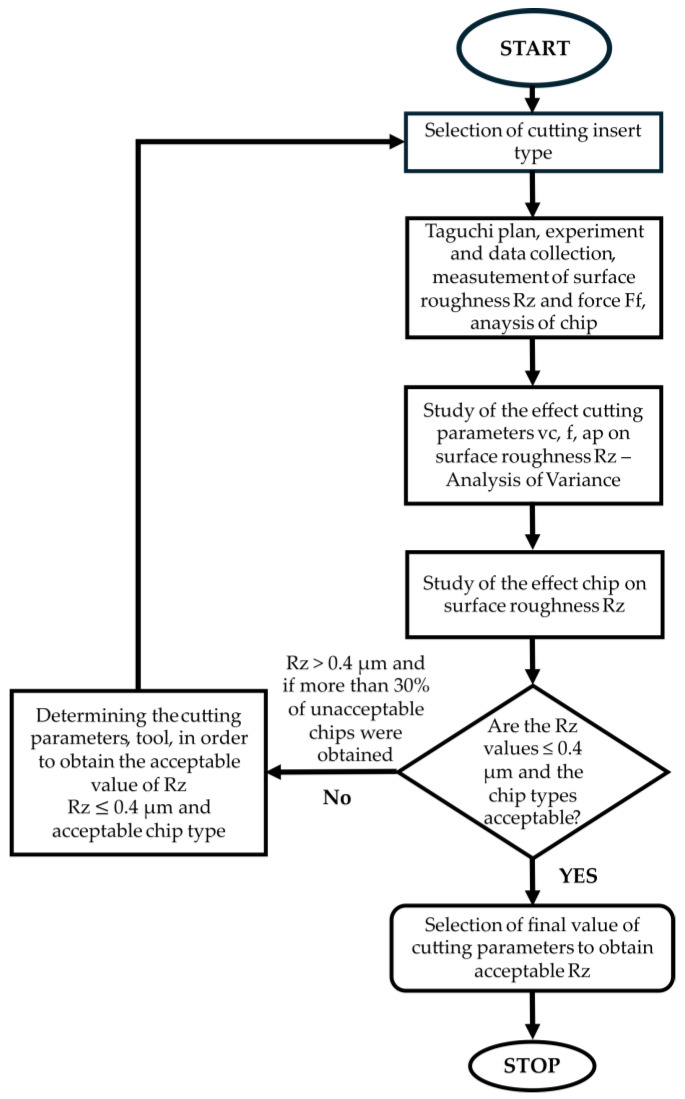
The algorithm for stepwise selection of acceptable turning parameters.

Based on the previous analysis of the Rz roughness results and the analysis of the shape and type of chips for both cutting depths, it was assumed that a cutting depth of *ap* = 0.1 mm provides more efficient machining. The algorithm for the results analysis and the sequence of selecting the turning process parameters to obtain a surface roughness of Rz ≤ 0.4 µm are presented below (Figure 14).

**Figure 15:**
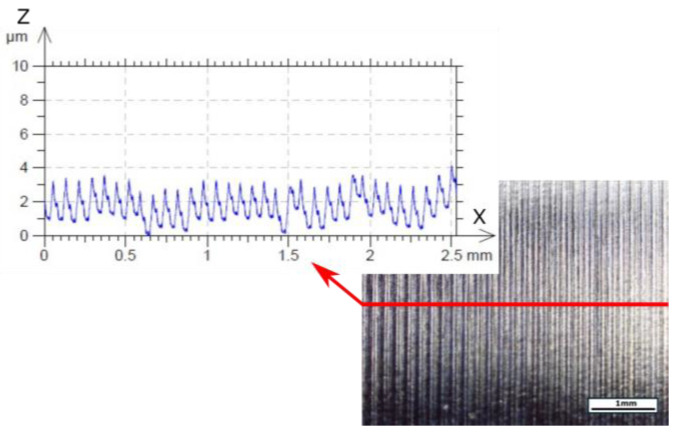
Photograph of machined surface with profile after turning using parameters such as *vc* = 70 m/min, *f* = 0.077 mm/rev, *ap* = 0.1 mm, and the TH tool.

**Figure 16:**
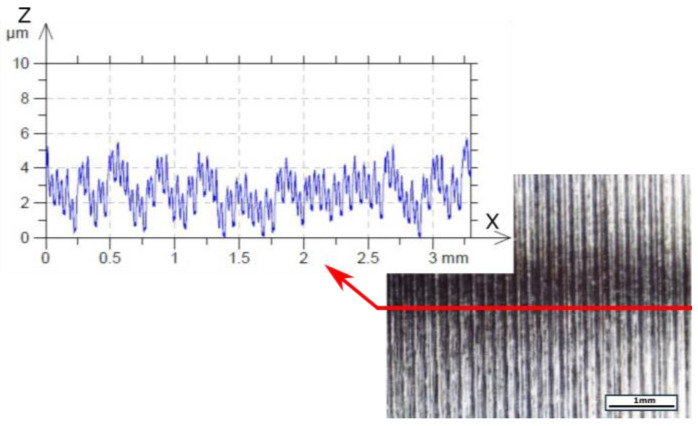
Photograph of machined surface with profile after turning using parameters such as *vc* = 130 m/min, *f* = 0.077 mm/rev, *ap* = 0.1 mm, and the TH tool.

The surface topography of the obtained surface with the most effective parameters of this turning process selected (*vc* = 70 m/min, *f* = 0.077 mm/rev, *ap* = 0.1 mm) is presented below (Figure 15). For comparison, the surface obtained at the maximum cutting speed is also presented (Figure 16).

## 4. Conclusions

The present study investigated the influence of cutting parameters and insert type on surface roughness (Rz) during turning of Inconel 625. The results enabled identification of the cutting conditions that ensured a surface finish of Rz ≤ 0.4 µm. The experiments also confirmed the high complexity of the Inconel 625 machining process, which is primarily attributed to its unfavorable thermal properties—most notably its low thermal conductivity (heat generated in the cutting zone is not effectively dissipated into the workpiece) and strong work-hardening tendency (leading to increased cutting forces, tool chatter, and compromised surface integrity in the form of micro-cracks, tearing, or smeared material). Therefore, it was advisable to apply cooling of the cutting zone using an efficient method such as MQL. The main conclusions can be summarized as follows:

- The best surface quality was obtained while using inserts with a 0.4 mm corner radius and an MF1 chip breaker.
- The cutting parameters that enabled achieving surface roughness Rz ≤ 0.4 µm were a cutting speed of 70 m/min, a feed rate of 0.077 mm/rev, and a depth of cut of 0.1 mm.
- Reduced cutting speed (70 m/min) contributed to lower heat generation, which promoted stable chip formation and protected the machined surface from damage.
- The tests confirmed that the thermal properties of the Inconel 625 superalloy significantly affect the stability of the cutting process and chip geometry, which in turn impacts the quality of the machined surface.

The proposed algorithm for selecting the parameter values acceptable for the adopted assumptions (Rz ≤ 0.4 µm) provides an innovative contribution to further research aimed at optimizing the machining of nickel–chromium superalloys. This is an important contribution given the enormous challenges associated with machining these superalloys. Future research should concentrate on narrowing ranges of cutting speeds (below 100 m/min) and employ advanced optimization approaches, such as response surface methodology or neural networks, in order to further improve surface finish while simultaneously reducing manufacturing costs and machining time. Additional analyses of chip morphology and machined surfaces using scanning electron microscopy (SEM) could provide further insights into the role of thermal-related phenomena in the machinability of Inconel 625.

## Figures and Tables

**Table 1:** Chemical composition of Inconel 625 superalloy [24].

Element	Ni	Cr	Mo	Fe	Nb	Co	Si	Mn	Ti	Al	C
wt. (%)	Min. 58	20–23	8–10	5	3.2–4.2	1	0.5	0.5	0.4	0.4	0.1

**Table 2:** Selected properties of Inconel 625 [5].

Property	Value
Mechanical	
Tensile strength (MPa)	1050
Elastic modulus (GPa)	205
Hardness (HB)	320
Poisson’s ratio	0.31
Elongation to break (%)	25–30
Thermophysical	
Thermal conductivity (W m^−1^ K^−1^)	10
Specific heat (J kg^−1^ K^−1^)	410
Density (g/cm^3^)	8.4

**Table 3:** Experimental test plan and average Rz surface roughness values.

	Machining Parameter			Surface Roughness Parameter—Mean Value of Rz (µm)		
Test No.	*vc* (m/min)	*f* (mm/rev)	*a_p_* (mm)	TS	CP	TH
1	70	0.077	0.1	0.16167	0.18500	0.17600
2	70	0.115	0.1	0.27233	0.29267	0.14367
3	70	0.154	0.1	0.28133	0.22567	0.38667
4	100	0.077	0.1	0.40133	0.25767	0.26600
5	100	0.115	0.1	0.49500	0.35833	0.40833
6	100	0.154	0.1	0.24633	0.39867	0.57433
7	130	0.077	0.1	0.51467	0.18967	0.24900
8	130	0.115	0.1	0.43833	0.39133	0.36733
9	130	0.154	0.1	0.58200	0.50633	0.39200
10	70	0.077	0.5	0.16167	0.18567	0.19767
11	70	0.115	0.5	0.27233	0.24900	0.18733
12	70	0.154	0.5	0.28133	0.25467	0.23467
13	100	0.077	0.5	0.40133	0.23833	0.12900
14	100	0.115	0.5	0.49500	0.41833	0.27800
15	100	0.154	0.5	0.24633	0.39900	0.37367
16	130	0.077	0.5	0.51467	0.18267	0.18567
17	130	0.115	0.5	0.43833	0.41367	0.43333
18	130	0.154	0.5	0.58200	0.61867	0.40367

**Table 4:** Mean values of Rz and standard deviation for the TS, CP, and TH tools, turning with the use of MQL.

	Mean Value of Rz (µm)			Standard Deviation		
Test No.	TS	CP	TH	TS	CP	TH
1	0.162	0.185	0.176	0.021	0.014	0.014
2	0.272	0.293	0.144	0.138	0.070	0.070
3	0.281	0.226	0.387	0.124	0.056	0.056
4	0.401	0.258	0.266	0.113	0.019	0.019
5	0.495	0.358	0.408	0.129	0.074	0.074
6	0.246	0.399	0.574	0.072	0.096	0.096
7	0.515	0.190	0.249	0.149	0.019	0.019
8	0.438	0.391	0.367	0.045	0.066	0.066
9	0.582	0.506	0.392	0.118	0.153	0.153
10	0.207	0.186	0.198	0.048	0.049	0.049
11	0.209	0.249	0.187	0.019	0.119	0.119
12	0.202	0.255	0.235	0.057	0.098	0.098
13	0.136	0.238	0.129	0.048	0.098	0.098
14	0.280	0.418	0.278	0.081	0.178	0.178
15	0.332	0.399	0.374	0.042	0.083	0.083
16	1.008	0.183	0.186	0.106	0.042	0.042
17	0.490	0.414	0.433	0.079	0.147	0.147
18	0.508	0.619	0.404	0.318	0.116	0.116

**Table 5:** Mean values of Rz and standard deviation for the TS, CP, and TH tools, dry turning.

	Mean Value of Rz (µm)			Standard Deviation		
Test No.	TS	CP	TH	TS	CP	TH
1	0.557	0.290	0.285	0.030	0.025	0.038
2	0.231	0.138	0.197	0.065	0.020	0.063
3	0.249	0.187	0.169	0.084	0.031	0.037
4	0.190	0.183	0.161	0.040	0.081	0.053
5	0.387	0.253	0.284	0.064	0.066	0.018
6	0.409	0.345	0.426	0.028	0.131	0.088
7	0.280	0.191	0.262	0.104	0.017	0.065
8	0.300	0.333	0.206	0.013	0.044	0.010
9	0.533	0.330	0.331	0.091	0.151	0.122
10	0.513	0.297	0.297	0.082	0.112	0.020
11	0.194	0.242	0.281	0.039	0.053	0.060
12	0.306	0.624	0.414	0.021	0.116	0.084
13	0.731	0.146	0.197	0.243	0.023	0.013
14	0.606	0.305	0.397	0.218	0.041	0.030
15	0.464	0.529	0.388	0.228	0.095	0.082
16	0.818	0.292	0.293	0.249	0.044	0.025
17	2.947	0.654	0.423	0.202	0.147	0.100
18	2.227	0.534	0.737	0.430	0.190	0.061

**Table 6:** Statistical results of ANOVA for Rz.

	CP				TH			
Source	DF	Adj SS	Adj MS	*p*-Value	DF	Adj SS	Adj MS	*p*-Value
*vc* (m/min)	2	0.0745	0.0372	0.010	2	0.0551	0.0275	0.032
*f* (mm/rev)	2	0.1230	0.0615	0.002	2	0.1126	0.0563	0.003
*ap* (mm)	1	0.0013	0.0013	0.627	1	0.0162	0.0162	0.125
Error	12	0.064	0.0053		12	0.0714	0.0059	
Total	17	0.263			17			

**Table 7:** Model summary for ANOVA.

	R-sq	R-sq(adj)
Rz_CP(*vc*,*f*,*ap*)	75.62%	65.46%
Rz_TH(*vc*,*f*,*ap*)	72.05%	60.40%

**Table 8:** The statistical results of ANOVA for *Ff*.

	CP				TH			
Source	DF	Adj SS	Adj MS	*p*-Value	DF	Adj SS	Adj MS	*p*-Value
*vc* (m/min)	2	188.3	94.2	0.450	2	134.4	67.2	0.119
*f* (mm/rev)	2	2587.3	1293.7	0.002	2	879.9	440.0	0.000
*ap* (mm)	1	10,743.5	10,743.8	0.000	1	12,137.3	12,137.3	0.000
Error	12	1323.5	110.3		12	315.9	26.3	
Total	17	14,843			17	13,467.5		

**Table 9:** Model summary for ANOVA.

	R-sq	R-sq(adj)
Ff_CP(*vc*,*f*,*ap*)	91.08%	87.37%
Ff_TH(*vc*,*f*,*ap*)	97.65%	96.68%

## Data Availability

The original contributions presented in this study are included in the article. Further inquiries can be directed to the corresponding author.

## References

[1] Singh B.J. (2022). Alloy 625: Microstructure, Properties and Performance.

[2] De Oliveira M.M., Couto A.A., Almeida G.F.C., Reis D.A.P., de Lima N.B., Baldan R. (2019). Mechanical Behavior of Inconel 625 at Elevated Temperatures. Metals.

[3] Pedroso A.F.V., Sousa V.F.C., Sebbe N.P.V., Silva F.J.G., Campilho R.D.S.G., Sales-Contini R.C.M., Jesus A.M.P. (2023). A Comprehensive Review on the Conventional and Non-Conventional Machining and Tool-Wear Mechanisms of INCONEL^®^. Metals.

[4] Liu E., An W., Xu Z., Zhang H. (2020). Experimental study of cutting-parameter and tool life reliability optimization in inconel 625 machining based on wear map approach. J. Manuf. Process..

[5] Parida A.K., Maity K. (2018). Comparison the machinability of Inconel 718, Inconel 625 and Monel 400 in hot turning operation. Eng. Sci. Technol. Int. J..

[6] Guo Q., Li D., Guo S., Peng H., Hu J. (2011). The effect of deformation temperature on the microstructure evolution of Inconel 625 superalloy. J. Nucl. Mater..

[7] Scendo M., Staszewska-Samson K., Danielewski H. (2021). Corrosion behavior of Inconel 625 coating produced by laser cladding. Coatings.

[8] Verdi D., Garrido M.A., Múnez C.J., Poza P. (2014). Mechanical properties of Inconel 625 laser cladded coatings: Depth sensing indentation analysis. Mater. Sci. Eng. A.

[9] Karmuhilan M., Kumanan S.A. (2022). Review on additive manufacturing processes of Inconel 625. J. Mater. Eng. Perform..

[10] Deshmukhe M., Raut Y., Joshi K. (2024). A review on fabrication of inconel alloys using different additive manufacturing techniques. AIP Conf. Proc..

[11] Subrahmanyam M., Nancharaiah T. (2020). Optimization of process parameters in wire-cut EDM of Inconel 625 using Taguchi’s approach. Mater. Today Proc..

[12] Tata N., Pacharu R.K., Devarakonda S.K. (2021). Multi response optimization of process parameters in wire-cut EDM on INCONEL 625. Mater. Today Proc..

[13] Jangali Satish G., Gaitonde V.N., Kulkarni V.N. (2021). Traditional and non-traditional machining of nickel-based superalloys: A brief review. Mater. Today Proc..

[14] Rathi N., Kumar P., Khatkar S.K., Gupta A. (2023). Non-conventional machining of nickel based superalloys: A review. Mater. Today Proc..

[15] Ezugwu E.O., Wang Z.M., Machado A.R. (1999). The machinability of nickel-based alloys: A review. J. Mater. Process. Technol..

[16] De Souza Ruzzi R., da Silva R.B., da Silva L.R.R., Machado A.R., Jackson M.J., Hassui A. (2020). Influence of grinding parameters on Inconel 625 surface grinding. J. Manuf. Process..

[17] Mohammadian N., Turenne S., Brailovski V. (2018). Surface finish control of additively-manufactured Inconel 625 components using combined chemical-abrasive flow polishing. J. Mater. Process. Technol..

[18] Alsoufi M.S., Bawazeer S.A. (2025). Predictive Modeling of Surface Integrity and Material Removal Rate in Computer Numerical Control Machining: Effects of Thermal Conductivity and Hardness. Materials.

[19] González M., Rodríguez A., Pereira O., de Lacalle L.N.L. (2025). Surface roughness evaluation when brushing heat-resistant alloy components. Int. J. Adv. Manuf. Technol..

[20] Yıldırım Ç.V. (2019). Experimental comparison of the performance of nanofluids, cryogenic and hybrid cooling in turning of Inconel 625. Tribol. Int..

[21] Akgün M., Demir H. (2021). Optimization of cutting parameters affecting surface roughness in turning of Inconel 625 superalloy by cryogenically treated tungsten carbide inserts. SN Appl. Sci..

[22] Prokes T., Mouralova K., Kovar J. (2016). Experimental evaluation on the quality of machined surface after turning of material Inconel 625. MM Sci. J..

[23] Makhesana M.A., Patel K.M., Krolczyk G.M., Danish M., Singla A.K., Khanna N. (2023). Influence of MoS_2_ and graphite-reinforced nanofluid-MQL on surface roughness, tool wear, cutting temperature and microhardness in machining of Inconel 625. CIRP J. Manuf. Sci. Technol..

[24] Machno M., Zębala W., Franczyk E. (2024). Optimization of turning of Inconel 625 to improve surface quality after finishing process. Materials.

[25] (2008). Geometrical Product Specifications (GPS)—Surface Texture: Profile Method—Terms, Definitions and Surface Texture Parameters (ISO 4287:1997/Cor 1:1998/Cor 2:2005).

[26] Liu L., Jiang X., Ying E., Sun Z., Geng D., Zhang D. (2025). Elliptical Ultrasonic Side Milling for Improved Surface Integrity and Fatigue Resistance of Thin-Walled Ti6Al4V Components. J. Zhejiang Univ. Sci. A.

[27] Chan L., Shyha I., Dreyer D., Hamilton J., Hackney P. (2017). Process optimisation for internal cylindrical rough turning of nickel alloy 625 weld overlay. Int. Sch. Sci. Res. Innov..

